# Leveraging the Fragment Molecular Orbital and MM-GBSA Methods in Virtual Screening for the Discovery of Novel Non-Covalent Inhibitors Targeting the TEAD Lipid Binding Pocket

**DOI:** 10.3390/ijms25105358

**Published:** 2024-05-14

**Authors:** Jongwan Kim, Haiyan Jin, Jinhyuk Kim, Seon Yeon Cho, Sungho Moon, Jianmin Wang, Jiashun Mao, Kyoung Tai No

**Affiliations:** 1 Bioinformatics and Molecular Design Research Center (BMDRC), Incheon 21983, Republic of Korea; haiyanjin@yonsei.ac.kr; 2 Department of Structural Biology, St. Jude Children’s Research Hospital, Memphis, TN 38105, USA; 3 The Interdisciplinary Graduate Program in Integrative Biotechnology & Translational Medicine, Yonsei University, Incheon 21983, Republic of Korea; yetyet123@yonsei.ac.kr (J.K.); jmwang113@hotmail.com (J.W.); jiashun_mao@yonsei.ac.kr (J.M.); 4 Baobab AiBIO Co., Ltd., Incheon 21983, Republic of Korea; sycho@baobabaibio.com (S.Y.C.); shmoon@baobabaibio.com (S.M.); 5 Department of Biotechnology, Yonsei University, Seoul 03722, Republic of Korea

**Keywords:** Hippo pathway, TEAD palmitoylation, virtual screening, molecular docking, fragment molecular orbital method, shape-based screening, luciferase reporter assay, drug discovery

## Abstract

The Hippo pathway controls organ size and homeostasis and is linked to numerous diseases, including cancer. The transcriptional enhanced associate domain (TEAD) family of transcription factors acts as a receptor for downstream effectors, namely yes-associated protein (YAP) and transcriptional co-activator with PDZ-binding motif (TAZ), which binds to various transcription factors and is essential for stimulated gene transcription. YAP/TAZ-TEAD facilitates the upregulation of multiple genes involved in evolutionary cell proliferation and survival. TEAD1–4 overexpression has been observed in different cancers in various tissues, making TEAD an attractive target for drug development. The central drug-accessible pocket of TEAD is crucial because it undergoes a post-translational modification called auto-palmitoylation. Crystal structures of the C-terminal TEAD complex with small molecules are available in the Protein Data Bank, aiding structure-based drug design. In this study, we utilized the fragment molecular orbital (FMO) method, molecular dynamics (MD) simulations, shape-based screening, and molecular mechanics–generalized Born surface area (MM-GBSA) calculations for virtual screening, and we identified a novel non-covalent inhibitor—BC-001—with IC_50_ = 3.7 μM in a reporter assay. Subsequently, we optimized several analogs of BC-001 and found that the optimized compound BC-011 exhibited an IC_50_ of 72.43 nM. These findings can be used to design effective TEAD modulators with anticancer therapeutic implications.

## 1. Introduction

The Hippo pathway was initially discovered in Drosophila in 1995 [1]. It is an evolutionarily conserved signaling pathway that controls organ size and maintains tissue homeostasis [2]. This signaling pathway is related to cancers [3], regenerative medicine [4,5], and neurodegenerative disorders [6,7]. Upstream of the Hippo pathway are core kinases, including MST1/2, MAP4K1–7, and LATS1/2, which bind to SAV1 and MOB1. The downstream regions of these core cascades are yes-associated protein (YAP) and transcriptional co-activator with PDZ-binding motif (TAZ) [7]. When the Hippo pathway is ‘on’, YAP and TAZ are phosphorylated by the activated core kinase cascades, promoting their sequestration (through interaction with 14-3-3 protein) or degradation (by the proteasome). On the contrary, when the Hippo pathway is ‘off’, unphosphorylated YAP and TAZ translocate into the nucleus, forming a complex with the transcriptional enhanced associate domain (TEAD) transcription factors, to drive the expression of target genes such as *Ctgf*, *Axl*, *Cyr61*, *Survivin*, *Sox2*, *Nanog*, and *Oct4*. Their expression is associated with cell proliferation and the inhibition of apoptosis, stemness, and pluripotency [7,8].

The dysregulation of the Hippo pathway has been implicated in the development of cancer and other diseases [9]. Amongst cancers, the pathway has been found to be involved in liver [10], breast [11], lung [12], pancreatic [13], and brain cancers [14]. The Hippo pathway has emerged as a promising target for cancer therapy because of its role in the development and progression of cancers, its potential to inhibit tumor growth and metastasis, and the development of targeted drugs that can inhibit this pathway [15,16].

**Figure 1:**
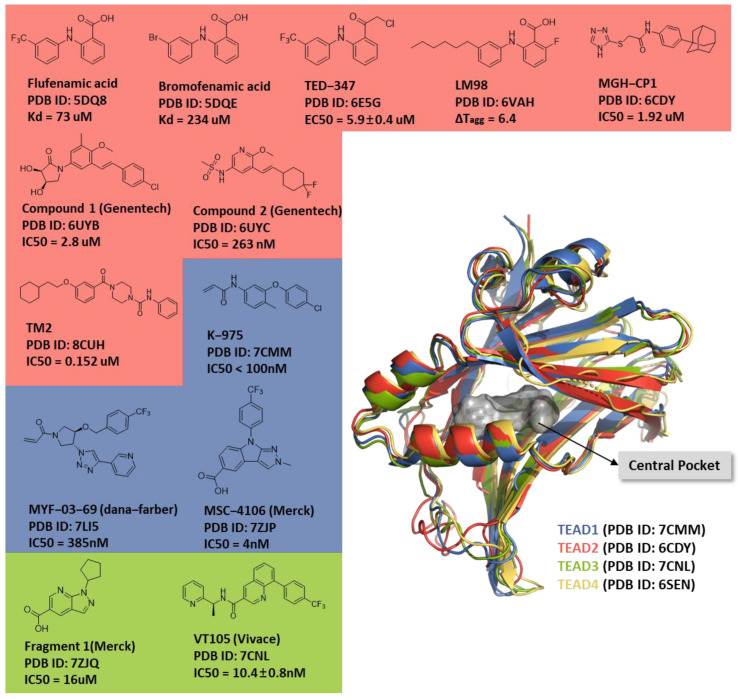
Superposition of TEAD1–4 and the chemical structure of reference compounds. Chemical structures of crystalline inhibitors and TEADs. Chemicals in the blue box indicate binding to TEAD1, red indicates binding to TEAD2, and green indicates binding to TEAD3. The right picture shows the superposition of TEAD1–4, with TEAD1 in blue, TEAD2 in red, TEAD3 in green, TEAD4 in yellow, and the central pocket depicted as the gray surface. TEAD, transcriptional enhanced associate domain; PDB, Protein Data Bank.

There are four families of TEAD transcription factors (TEAD1–4) with highly conserved sequences and structural domains. They all contain a yap-binding domain (YBD, C-terminal) and a DNA-binding domain (N-terminal) [17]. TEAD YBDs are popular binding domains of several partner cofactor proteins, including Hippo-dependent co-activators YAP and TAZ and Hippo-independent co-activators VGLL1–4 and FAM181 A/B [18]. According to several reported crystal structures of the YAP-TEAD complex, there are three interfaces between YAP and TEAD: β-strand (interface 1), α-helix (interface 2), and Ω-loop (interface 3) [19,20]. The YBD of the TEAD family also contains a highly conserved autolipidation pocket for becoming functional; this pocket is on a conserved cysteine residue located at the entrance of the central pocket [21,22]. In 2015, using X-ray crystallization, Pobbati et al. first identified flufenamic acid (a non-steroidal anti-inflammatory drug) and bromofenamic acid as molecules that bind to the central pocket of TEAD2 [23]. Since then, several crystal structures of the inhibitors and the TEAD complex have been released in the Protein Data Bank (https://www.rcsb.org), as shown in Figure 1.

Two strategies are used to target the downstream Hippo pathway. The first involves disruption of the protein–protein interactions (PPIs) between YAP and TEAD. Interfaces 2 and 3 appear to be better target sites for inhibitors of the YAP-TEAD interaction [24]. An early strategy was to design YAP to mimic peptide–drug binding in the Ω-loop pocket [25,26]. Recently, the α-helix mimic peptide has also been reported [27]. In 2012, Liu et al. identified verteporfin as a small molecule that inhibits the YAP-TEAD association [28]. Fragment 1 exhibited submicromolar activity and was identified using fragment-based and computational modeling approaches [29]. Our group previously reported a BY series of inhibitors that can bind to the YAP-TEAD interface 2, using computational virtual screening [30]. Recently, Novartis confirmed the first nanomolar small molecules that bind to the YAP-TEAD interface 3, using X-ray crystallization [31].

The second strategy involves inhibiting TEAD palmitoylation by binding to the central pocket. Three small-molecule inhibitors of TEADs are currently being evaluated in Phase I clinical trials; however, their chemical structures are unknown. IK930 was obtained from Ikena Oncology (NCT05228015), VT-3989 from Vivace Therapeutics (NCT04665206), and IAG933 from Novartis (NCT04857372). IK930 and VT-3989 are TEAD auto-palmitoylation inhibitors [32,33], while there is yet no information on IAG933. Pocket inhibitors can be divided into two classes: reversible and irreversible. A covalent warhead group, such as acrylamide, can irreversibly bind to conserved cysteine residues (Cys359 in TEAD1, Cys380 in TEAD2, Cys368 in TEAD3, and Cys367 in TEAD4), while the rest of the structure of the compound is a hydrophobic moiety. Non-covalent compounds can also be divided into two parts, hydrophilic and hydrophobic, based on the characteristics of the central pocket. Since then, only two research studies have identified TEAD inhibitors using computational approaches [34,35]. However, while one study did not measure the activity using an in vitro assay, the hit compound of the other study had low potency.

**Figure 2:**
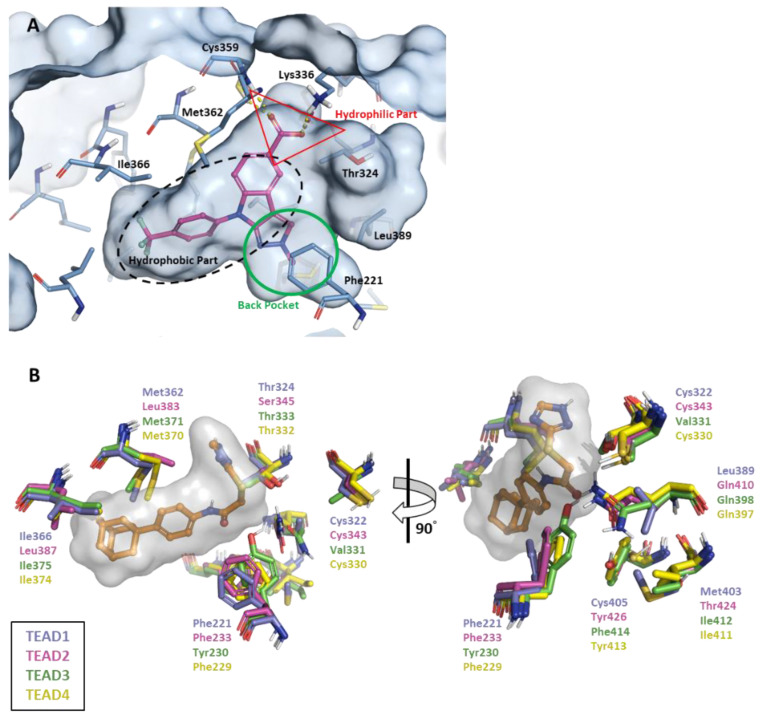
Characteristics of the TEAD central pocket. (**A**) Crystal structure of the MSC-4106 complex with TEAD1 and the three pockets. (**B**) The different residues of TEAD1–4 around the central pocket. TEAD1 residues are represented in blue sticks, TEAD2 in pink, TEAD3 in green, TEAD4 in yellow, and MGH-CP1 in orange. TEAD, transcriptional enhanced associate domain.

TEADs have four paralog families. Sequence alignment of the YBD of the four families showed a highly conserved hydrophobic pocket around the central pocket [36]. The central pocket comprises three major parts: hydrophilic, hydrophobic, and back pockets. First, the hydrophilic portion was positioned at the entrance of the central pocket (Figure 2A). Two key residues are involved in this: cysteine (Cys359 in TEAD1, Cys380 in TEAD2, Cys368 in TEAD3, and Cys367 in TEAD4) and lysine (Lys336 in TEAD1, Lys357 in TEAD2, Lys345 in TEAD3, and Lys344 in TEAD4). The conserved cysteine residue covalently bonds to palmitate and several covalent inhibitors like K-975 [37] and also contributes to hydrogen bonding to the hydrophilic moiety of non-covalent inhibitors. The lysine residue is sometimes reported to be covalently bonded to myristate [38], but it contributes the most to hydrogen bonding with the inhibitors. Noland et al. reported that the C380A and K357A mutant TEAD2 proteins lost their overall protein expression [21]. Second, the hydrophobic pocket occupied by the palmitate alkyne chain contains several highly conserved hydrophobic residues, such as Phe, Leu, Val, Met, and Ile. Despite this, there is a possibility of finding different residues, such as Tyr230, in TEAD3 (Figure 2B). Finally, the back pocket is slightly complicated and was found recently [39,40,41]. There are six different residues in the back pocket of TEAD1–4, as shown in Figure 2B, including hydrophobic and hydrophilic residues. Therefore, the back pocket may be an essential part in the design of selective inhibitors of a unique TEAD family. However, according to the volume analysis of the central pocket, this pocket has remarkable plasticity to the ligand; therefore, based on the steric clash issue, the design of selective ligands is unlikely [36]. Here, we focused on the hydrophilic and hydrophobic parts to identify non-covalent pan-TEAD inhibitors.

In this study, we aimed to understand the characteristics of the central pocket of TEAD by employing the fragment molecular orbital (FMO) method and molecular dynamics (MD) simulations of four TEAD complex crystal structures with specific reference compounds (MGH-CP1, Compound 2, MSC-4106, and VT-105), known as non-covalent inhibitors in these structures. Our analysis revealed that hydrophobic interactions predominantly contribute to the binding interaction energy, with minimal non-bonding interactions, such as hydrogen bonds and electrostatic interactions. To streamline our virtual screening process, we incorporated shape-based screening, which can not only refer to the shape of the binding pocket cavity, but also decrease the computational cost. Furthermore, to account for the significant solvation effect in lipid pockets, we utilized the molecular mechanics–generalized Born surface area (MM-GBSA) calculation with a single conformer approach to accelerate the computation of binding free energy predictions between the protein and ligand [42,43]. This approach led us to identify BC-001 as a promising hit compound that exhibited micromolar activity in the TEAD reporter assay.

After optimizing and synthesizing several compounds, we discovered BC-011 with an IC_50_ of 72.43 nM in the luciferase reporter assay. Moreover, BC-011 inhibited H226 cancer cell viability at an IC_50_ level of 488.5 nM and downregulated the expression of the target genes *Ctgf* and *Cyr61*.

## 2. Results

### 2.1. FMO Analysis of TEAD Complexes with Reference Compounds: Insights into Binding Interactions

**Figure 3:**
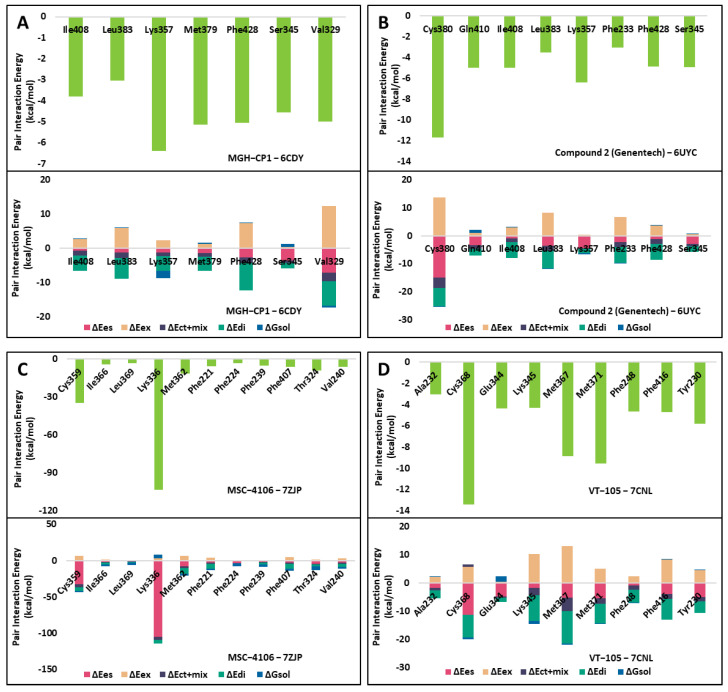
Fragment Molecular Orbital results of the crystal structures of the four reference compounds. (**A**) MGH-CP1, (**B**) Compound 2, (**C**) MSC-4106, and (**D**) VT-105. The pair interaction energies of the significant residues are shown in the top panel of each figure. The bottom panel of each figure represents the pair interaction energy decomposition analysis of these critical interactions. The electrostatic, exchange repulsion, and charge transfer with higher-order mixed term, dispersion, and solvation energy terms are shown in pink, salmon, navy, green, and blue, respectively.

We conducted FMO calculations on the four crystal structures of non-covalent inhibitors (MGH-CP1, Compound 2, VT-105, and MSC-4106) complexed with TEAD, with IC_50_ values of 1920 nM, 263 nM, 10.4 nM, and 4 nM, respectively. In the TEAD2 complex with MGH-CP1, Lys357 emerged as the most significant residue, with a pair interaction energy (PIE) of −6.385 kcal/mol. The dispersion and exchange repulsion energies were predominant, as shown in Figure 3A and Table S1. For the Compound 2 complex, Cys380 displayed the lowest energy, at −11.701 kcal/mol, with an electrostatic term of −14.899 kcal/mol, due to hydrogen bonding with sulfonyl oxygen atoms (Figure 3B and Table S2). In the MSC-4106 complex, Lys336 (−103.158 kcal/mol) and Cys359 (−34.712 kcal/mol) exhibited lower energies, primarily driven by electrostatic interactions involving hydrogen bonds and salt bridges (Figure 3C and Table S3). Similar results were observed for TEAD3, with Cys368 being the most significant residue, at −13.387 kcal/mol, involving hydrogen bonding with the carbonyl of VT-105 (Figure 3D and Table S4). Overall, these findings indicated that one or two non-bonding interactions occur at the entrance of the central pocket, whereas the deep pocket is predominantly characterized by hydrophobic interactions.

### 2.2. MD Analysis of TEAD Complexes with Reference Compounds: Unveiling Structural Insights

**Figure 4:**
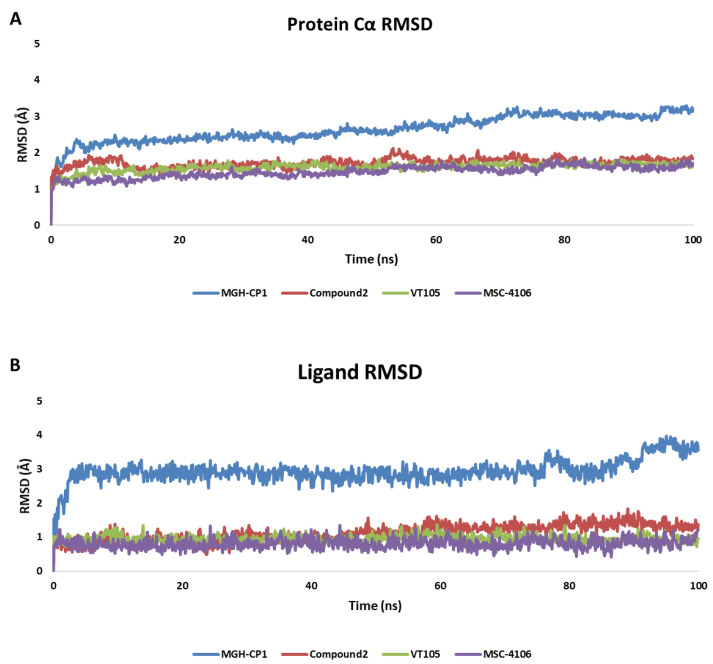
Average RMSD calculations of three replicas. (**A**) The protein Cα and (**B**) ligand RMSDs of the four reference compounds. RMSD, root mean square deviation.

**Figure 5:**
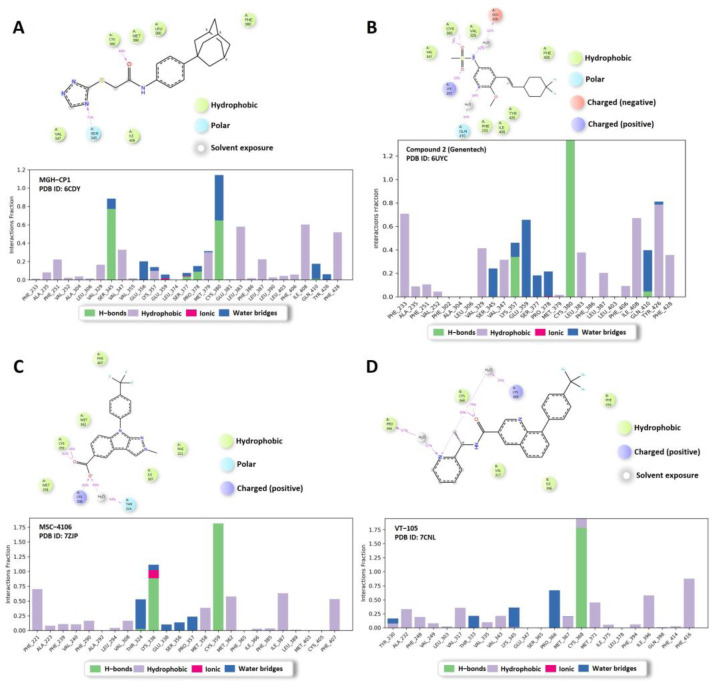
Molecular dynamics simulation analysis of the four reference structures. (**A**) MGH-CP1, (**B**) Compound 2, (**C**) MSC-4106, and (**D**) VT-105. Protein–ligand contacts of the 2D chemical structures are shown in the top picture of each figure. The 2D summary shows interactions that occur in more than 30.0% of the simulation time. The bottom panel of each figure represents the histogram of the protein–ligand interaction fraction.

We conducted MD simulations of the same crystal structures as the non-covalent compounds. Among these, MGH-CP1 displayed relatively high instability, correlating with its submicromolar IC_50_ (the average RMSD of three replicas is shown in Figure 4). The protein Cα RMSD (Figure 4A) and ligand RMSD (Figure 4B) of MGH-CP1 showed an unstable fluctuation graph compared to the other three ligands. Figure 5 illustrates the representative protein–ligand contacts and interaction fractions of the four compounds. Hydrogen bond maintenance between the carbonyl group of MGH-CP1 and Cys380 was only 48% (Figure 5A), corresponding to its micromolar IC_50_. In contrast, the other three compounds displayed significantly higher percentages of protein–ligand contacts: Compound 2 maintained 79% with Cys380 (TEAD2) through the sulfonyl group (Figure 5B), MSC-4106 maintained 99% with Cys359 (TEAD1) via the carboxyl group (Figure 5C), and VT-105 (TEAD3) maintained 99% with Cys368 through the carbonyl group (Figure 5D). The protein–ligand histogram revealed that hydrophilic interactions (H-bonds and ionic interactions) and water bridges were concentrated on residues at the entrance of the central pocket, forming a hydrophilic part. The hydrophobic interaction fractions were distributed within the pocket, constituting the hydrophobic and back-pocket regions. This MD analysis of the four stabilized complexes reaffirmed that the central pocket can be categorized into a hydrophilic region at the pocket entrance and a hydrophobic region deeper within the pocket.

### 2.3. Structure-Based Virtual Screening Using MM-GBSA for Tool Compound Identification

**Figure 6:**
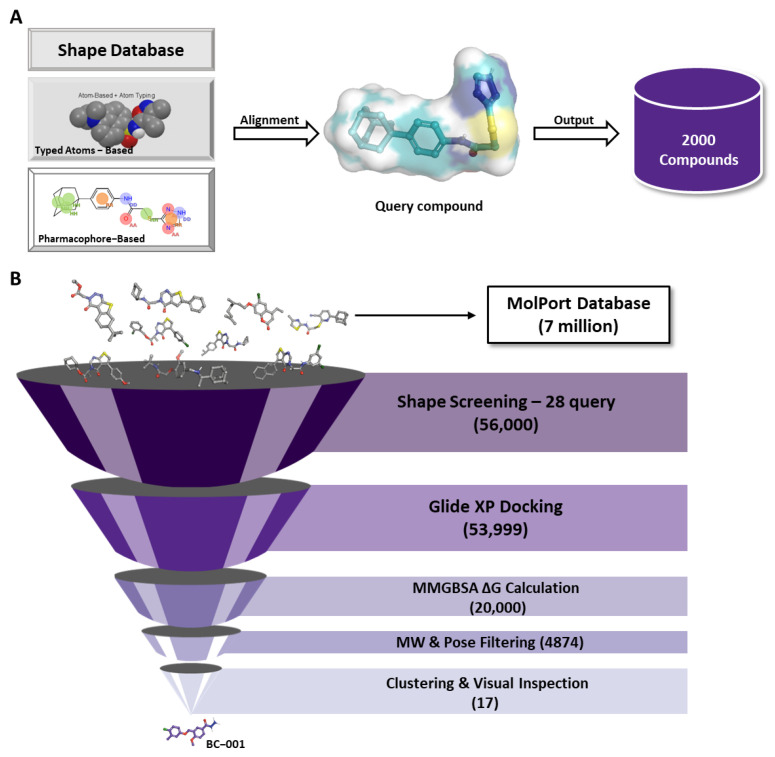
Structure-based virtual screening. (**A**) Shape-based screening workflow. The figure shows the shape-based screening workflow for each query compound. (**B**) Protocol for structure-based virtual screening of the transcriptional enhanced associate domain modulator.

**Figure 7:**
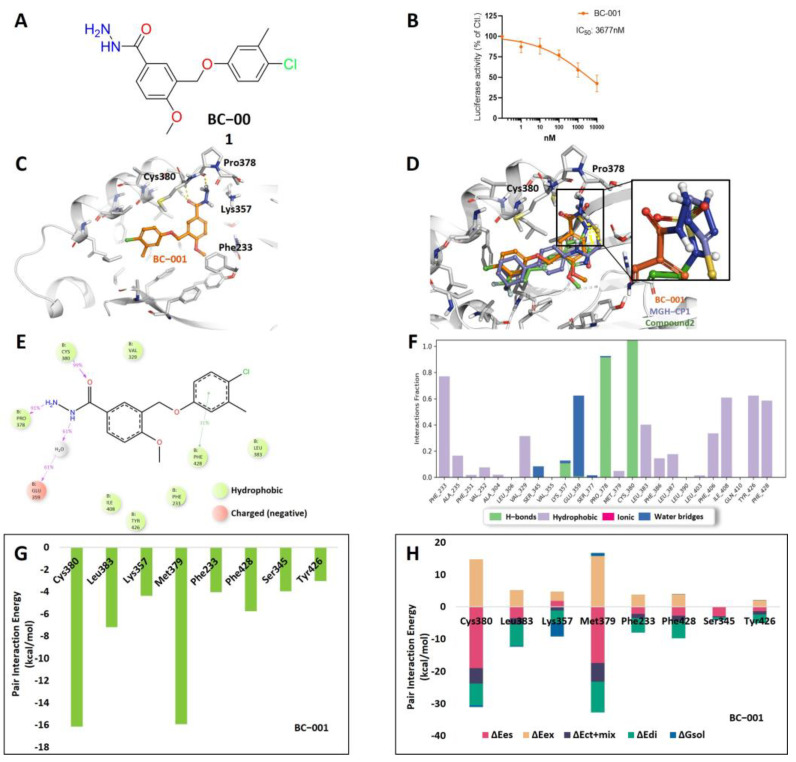
Luciferase reporter assay and docking structure of BC-001. (**A**) Chemical structure of BC-001. (**B**) TEAD luciferase reporter activity observed in MCF7 cells with 1 nM to 10 μM of BC-001 (*n* = 2). (**C**) Docking pose of BC-001 in the TEAD2 central pocket. The ligand is represented in orange. (**D**) Overlay structures of the three ligands, with BC-001 in orange, MGH-CP1 in blue, and Compound 2 in green. The hydrophilic part has been magnified. (**E**) Protein–ligand contacts of the BC-001 2D chemical structure. The 2D summary shows interactions that occur in more than 30.0% of the simulation time. (**F**) Histogram of the protein–ligand interaction fraction. (**G**) PIEs of the significant residues involved in the binding of TEAD2 with BC-001. (**H**) PIE decomposition analysis of these critical interactions. The electrostatic, exchange repulsion, and charge transfer with higher-order mixed term, dispersion, and solvation energy terms are shown in pink, salmon, navy, green, and blue, respectively. TEAD, transcriptional enhanced associate domain; PIE, pair interaction energy.

We selected 28 unique reference compounds capable of binding to the central pocket as query compounds for shape-based screening. Prior to conducting shape-based screening, we performed enrichment calculations to optimize the screening parameters (Figure S1). Initially, we used the Molport database (https://www.molport.com/), which contains seven million commercial screening compounds, to create a shape data file for graphics processing using the Maestro program. We created two specific shape databases: one based on pharmacophores and the other on typed atoms. Each query compound was retained to 2000 aligned compounds from both databases (Figure 6A). This process was repeated 28 times for 28 query compounds, resulting in 56,000 screened compounds based on their shape. Considering that the pocket volume of the MGH-CP1 complex with TEAD2 (PDB ID: 6CDY) was larger than that of other crystal structures of non-covalent inhibitors, we employed the 6CDY structure to generate a grid for molecular docking simulations. As shown in Figure 6B, 56,000 compounds were identified using shape-based screening. These compounds were subjected to molecular docking using the Glide XP method in the Schrödinger program, resulting in the identification of 53,999 ligands. Subsequently, we performed MM-GBSA ΔG values calculations for these compounds to more accurately rank potential ligands. To minimize calculation costs and enable MM-GBSA calculations for 53,999 ligands, we conducted MM-GBSA calculations using a single conformer approach with an implicit solvation model in Prime [42,43]. We ranked the MM-GBSA ΔG values and retained 20,000 compounds with absolute values lower than those of reference compounds. Ligands with molecular weights smaller than 245 g/mol were removed because we aimed to maintain a minimum molecular weight considering drug-likeness. If the molecular weight of a structure falls below that of palmitic acid (243 g/mol), a TEAD substrate, it may not fit well into the existing pocket or may be considered too fragment-like, leading to its removal. The Schrödinger pose filter module was utilized to filter poses that satisfied the fundamental interactions. After clustering and visual inspection, 17 virtual hits were selected for further validation using in vitro assays (Figure S2). Ultimately, we identified compound BC-001 (Figure 7A), which demonstrated an IC_50_ of 3677 nM in the TEAD reporter luciferase assay (Figure 7B).

### 2.4. Computational Analysis of the Compound BC-001

The carbonyl group of BC-001 formed a robust hydrogen bond with the side chain Cys380 SH and backbone NH in the docking pose of BC-001 (Figure 7C). This hydrogen bond was maintained for 99% of the simulation time during the 100 ns MD simulation (Figure 7E,F). Additionally, the hydrazine group at the head of BC-001, which acts as a hydrophilic group, interacts with the CO backbone of Pro378 through a hydrogen bond (Figure 7C). This interaction remained stable with a 91% contact rate during the simulation (Figure 7E,F). Compared to the reference compounds, the carbonyl group of BC-001 occupied the same position as the sulfonyl group in Compound 2 and the carbonyl groups in MSC-4106 and VT-105. Furthermore, the hydrazine group of BC-001 was aligned with the tetrazole group of MGH-CP1 (Figure 7D). In the FMO results, Cys380 and Met379 exhibited the lowest PIEs, owing to electrostatic interactions (Figure 7G,H and Table S5). According to the FMO method, fragmentation of the protein backbone occurs at the bond between the alpha and carbonyl carbon atoms. Therefore, in Pro378, the CO backbone may belong to a neighboring residue, Met379. The hydrophobic regions of BC-001 interact with the deep pocket, particularly around Val329, Phe428, Phe233, and Ile408 (Figure 7C). The PIEs of Phe428 and Phe233 were lower than −3 kcal/mol and were mainly distributed through the dispersion energy (Figure 7G,H and Table S5). These findings are consistent with the results of the analysis of the reference structures mentioned above (Figure 3, Figure 4 and Figure 5). Collectively, these in silico and in vitro results confirmed that BC-001 is a potent compound-binding site within the TEAD central pocket.

### 2.5. Structure–Activity Relationship Study of the Hit Compound BC-001, for Optimization

**Figure 8:**
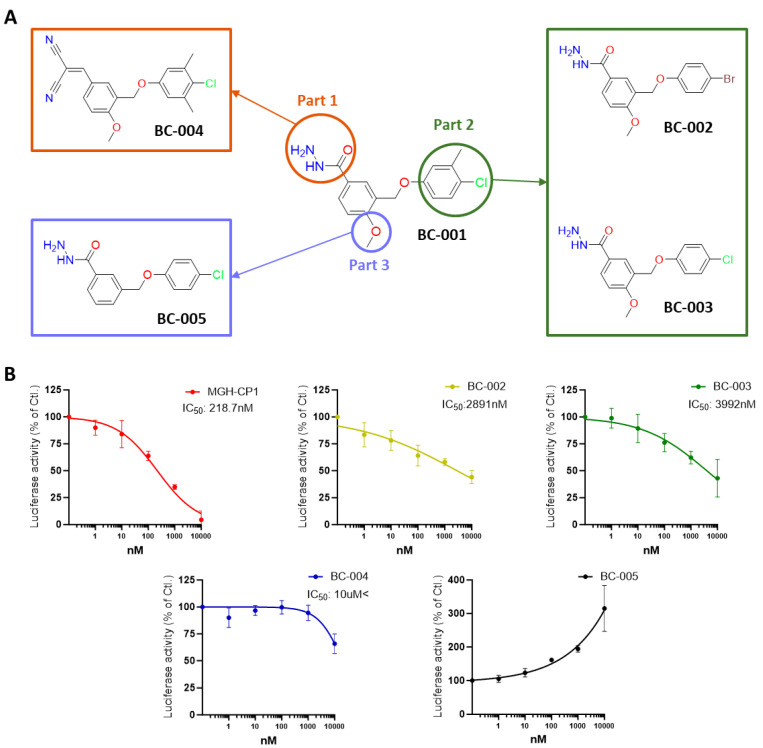
Structure–activity relationship of the hit compound BC-001 and reporter luciferase assay. (**A**) Chemical structures obtained upon substitution of three parts from BC-001. (**B**) Transcriptional enhanced associate domain reporter luciferase activity was observed in MCF7 cells treated with 1 nM to 10 μM of MGH-CP1, BC-001, BC-002, BC-003, BC-004, and BC-005 (*n* = 2).

To investigate whether BC-001 was the true hit compound, we selected five compounds from the clustering analysis of virtual screening that shared the same scaffold as BC-001. In Part 1 (Figure 8A), when the malononitrile group replaced the hydrazine group, the resultant BC-004 did not exhibit any activity in the reporter assay (Figure 8B). This indicates the necessity of hydrazine binding in this pocket area to maintain cellular activity. In Part 2 (Figure 8A), removing the methyl group (BC-003) or substituting chlorine with bromine (BC-002) retained the same activity as BC-001 (Figure 8B), confirming that BC-001 was the definitive hit compound. Finally, in Part 3 (Figure 8A), upon removal of the methoxy group from BC-003, reporter luciferase activity disappeared and appeared to increase.

**Figure 9:**
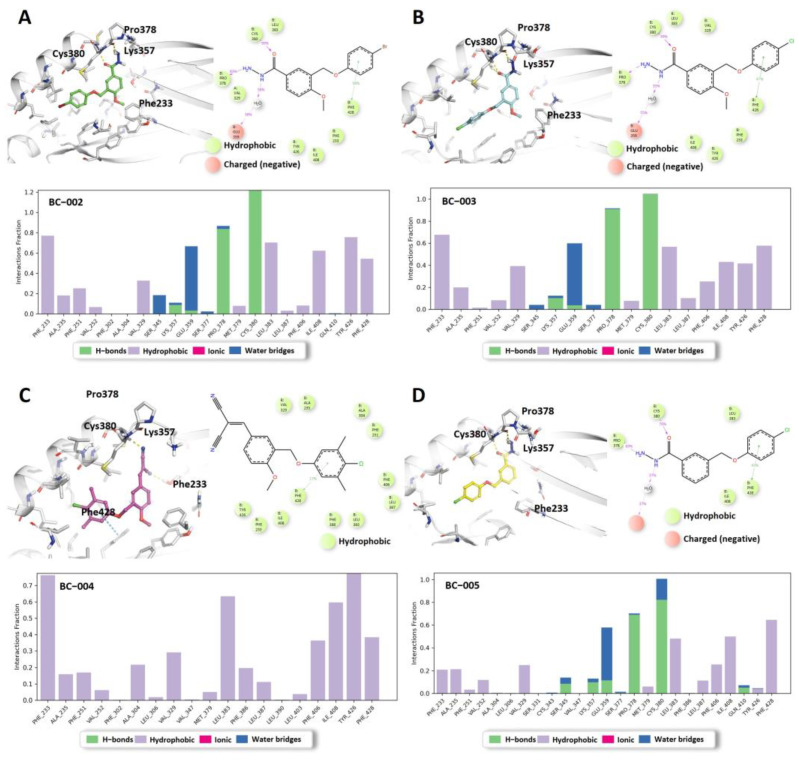
In silico analysis of BC-002, BC-003, BC-004, and BC-005. (**A**) Left top: docking structure of BC-002 with TEAD2, with the ligand represented in green; right top: protein–ligand contacts of BC-002. These represent interactions that occur in more than 30.0% of the simulation time. Bottom: histogram of the protein–ligand interaction fraction. (**B**) Left top: docking structure of BC-003 with TEAD2, with the ligand represented in light blue; right top: Protein–ligand contacts of BC-003. These represent interactions that occur in more than 30.0% of the simulation time. Bottom: histogram of the protein–ligand interaction fraction. (**C**) Left top: docking structure with TEAD2 of BC-004, with the ligand represented in pink; right top: protein–ligand contacts of BC-004. Bottom: histogram of the protein–ligand interaction fraction. (**D**) Left top: docking structure of BC-005 with TEAD2, with the ligand represented in yellow; right top: protein–ligand contacts of BC-005. Bottom: histogram of the protein–ligand interaction fraction.

Docking studies of BC-002 and BC-003 revealed the same binding mode as that of BC-001; the carbonyl group formed a hydrogen bond with Cys380, and the hydrazine group interacted with Pro378 (Figure 9A,B). Furthermore, protein–ligand contacts from the MD simulations of BC-002 and BC-003 mirrored the results obtained for BC-001; carbonyl and hydrazine groups formed hydrogen bonds with more than 80% contact, and these interactions were robust throughout the simulation (Figure 9A,B). The docking structure of BC-004 indicated that the malononitrile group formed a hydrogen bond with the critical residue, Cys380 (Figure 9C), suggesting that the malononitrile group could replace the hydrazine group. However, after the 100 ns MD simulation, the hydrophilic interactions at the entrance of the central pocket disappeared, especially in the histogram panel, with only hydrophobic interactions remaining. The docking and MD simulation results for BC-005 (Figure 9D) suggested that the compound might bind within the central pocket; however, the reporter luciferase activity increased. We hypothesized that BC-005 might be a potential TEAD activator, similar to the TEAD activators using the quinolinol scaffold reported by Pobbati et al. [44]. However, it remains unclear why the absence of a methoxy group leads to an increased activity in the reporter assay.

### 2.6. Retrospective Prediction of Binding Free Energy Using Post-MD MM-GBSA Average Values

**Figure 10:**
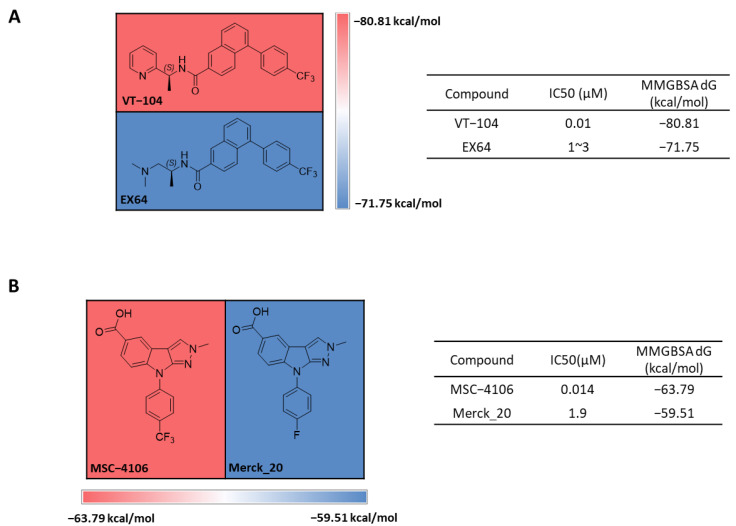
Retrospective prediction heatmap of post-MD MM-GBSA ΔG average values of Vivace and Merck compounds. Heatmap of the average post-MD MM-GBSA ΔG after a 100 ns simulation, (**A**) for Vivace and (**B**) for Merck compounds. MD, molecular dynamics; MM, molecular mechanics; GBSA, Generalized Born Surface Area.

We retrospectively predicted several compounds that share the same scaffold from the Vivace [45] (WO202097389A1) and Merck [40] groups (WO2021224291A1) using post-MD MM-GBSA [46] analysis (Figure 10). To evaluate the variety of samples in the 100 ns simulation, we utilized frames at 10, 20, and 50 ns, as well as the most stable frame at 10 ns, to calculate their post-MD MM-GBSA ΔG average values. We discovered that each of the sampling groups had a similar pattern when compared to sampling from the whole frames of 100 ns (Figure S3). Replacing pyridine with dimethylamine in VT-104 to create EX64 decreased IC50 by over 100-fold, and the post-MM-GBSA ΔG absolute values decreased correspondingly (Figure 10A). Replacing the trifluoromethyl group with fluorine caused a decrease in IC50, which was also reflected in the post-MM-GBSA ΔG absolute values (Figure 10B). These results demonstrated that the predicted ΔG values from the post-MD-MM-GBSA agree with experimental IC50 values.

### 2.7. Optimizing and Synthesizing the BC-001 Scaffold: Enhancing Binding Affinity and Biological Activity

**Figure 11:**
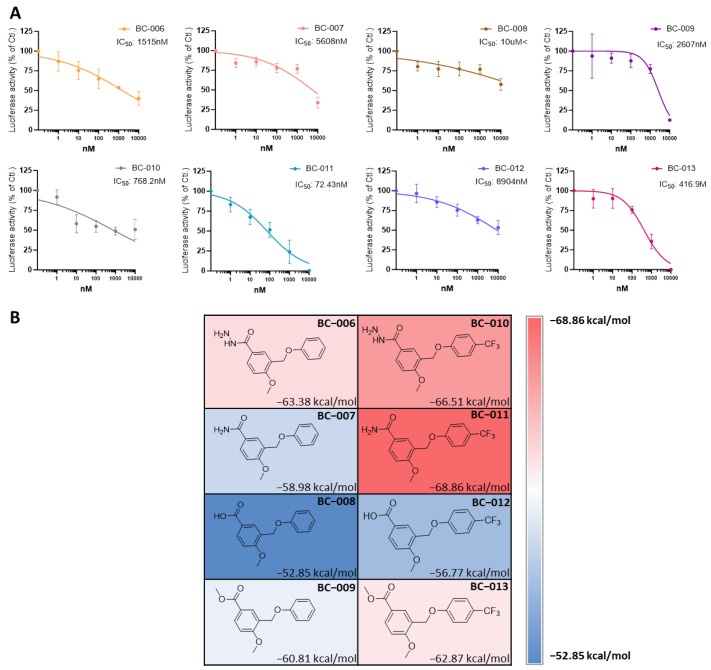
Reporter luciferase assay of compounds and heatmap of post-MD MM-GBSA ΔG. (**A**) TEAD reporter luciferase activity was observed in MCF7 cells treated with 1 nM to 10 μM of BC-006 to BC-013 (*n* = 2). (**B**) Heatmap of the average post-MD MM-GBSA ΔG after a 100 ns simulation, from BC-006 to BC-013. MM, molecular mechanics; GBSA, Generalized Born Surface Area; TEAD, transcriptional enhanced associate domain.

We further optimized and synthesized the BC-001 scaffold and validated the compounds using in vitro assays (Table 1 and Figure 11A). Initially, when R_2_ was fixed as hydrogen, compounds BC-006–BC-009 were obtained. BC-006 exhibited the most potent activity, with the hydrazine group as the R_1_ substituent. The activity diminished only when the R_1_ group was replaced with a hydroxyl group. Subsequently, compounds BC-010–BC-013 were synthesized, and their efficacy was restored by replacing trifluoromethyl with hydrogen at the R_2_ position. Trifluoromethyl is an essential moiety for enhancing binding affinity, as supported by various TEAD inhibitor scaffolds [39,40,45]. In particular, BC-011 displayed an IC_50_ of 72.43 nM, featuring an amine head group. In the heatmap of the eight structures from BC-006 to BC-013 (Figure 11B), the trend in ΔG values indicated that the trifluoromethyl group and methoxy were crucial functional groups contributing to hydrophobic interactions. The compounds with hydrazine and amine head groups displayed higher MM-GBSA ΔG absolute values than those with other functional groups (Figure 11B). However, establishing a direct correlation between post-MD MM-GBSA results and in vitro assays is challenging. This discrepancy is attributed to the cell-based conditions of the reporter assay, in which compound potency accounts for membrane permeability and solubility [47].

### 2.8. Cell Viability and TEAD Target Gene Expression Assay of BC-010, BC-011, and BC-013

**Figure 12:**
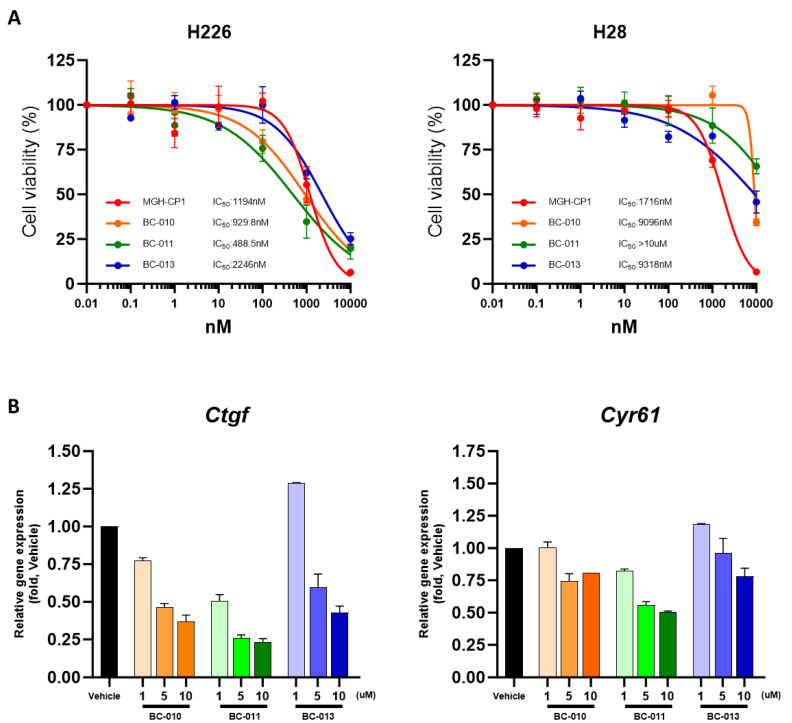
Cell viability assay and mRNA expression. (**A**) Cells were treated with various concentrations of MGH-CP1, BC-010, BC-011, and BC-013 for 144 h (*n* = 3). (**B**) The expression levels of target genes (*Ctgf* and *Cyr61*) in H226 cells incubated for 24 h in the presence of BC-010, BC-011, and BC-013, at concentrations of 1, 5, and 10 μM. The mRNA expression levels were measured using qRT-PCR (*n* = 3).

We further investigated the effects of BC-010, BC-011, and BC-013 on cancer cell viability and TEAD target gene expression. In the cell viability assay, BC-011 demonstrated the ability to inhibit H266 cancer cells with an IC_50_ level of 488.6 nM. However, in the H28 cancer cell line, BC-011 did not show any inhibitory effects on viability (Figure 12A). In the mRNA expression assay, the TEAD target genes, *Ctgf* and *Cyr61* were significantly downregulated by BC-011 (Figure 12B). These results indicated that BC-011 not only inhibits TEAD at the cellular level, but also decreases the expression of TEAD target genes.

## 3. Discussion

Targeting the Hippo-YAP-TEAD signaling pathway in various cancer types has emerged as a prominent area of interest in drug discovery. Initial research in this area concentrated on designing PPI inhibitors to disrupt the YAP-TEAD interaction. However, several challenges associated with PPI inhibitors, including limited efficacy and specificity, pose significant obstacles in developing highly potent drug candidates. Recently, the Novartis group successfully unveiled a series of exceptionally effective PPI inhibitors targeting the YAP-TEAD interface [31]. Consequently, many scientists have shifted their focus towards investigating the TEAD palmitoylation site as a promising novel binding pocket for the discovery of new inhibitors. Fortunately, the crystal structure of the TEAD holoform required for structure-based drug design has already been reported. However, the mode of action of central pocket inhibitors remains unclear. To overcome this hurdle and gain insights into the rational design of central pocket modulators, we employed a structure-based drug design approach and multiple computational methodologies to identify novel candidates for targeting the TEAD central pocket.

Central pocket inhibitors have a unique binding interaction pattern with TEAD because of the hydrophobic environment surrounding the central pocket located in the middle of the protein. There are only a few hydrophilic interactions, mainly occurring at the pocket’s entrance. We observed the same in the FMO study, which is extremely useful for quantitative understanding of molecular interactions and MD simulations of the reference compounds carried out in our study as well. The analysis of the reference compounds (MGH-CP1, 2, VT-105, and MSC-4106) using FMO highlighted the important residues (e.g., Lys357 and Cys380) and how they interacted with these compounds, with an emphasis on various energy components, particularly the hydrophobic effect represented by the dispersion. The results of the MD simulations illustrated how these simulations can identify the stability of these complexes and the structural changes that occur over time. These analyses of the structure-based binding sites demonstrated the unique characteristics of the central pocket.

Due to the unique nature of the central pocket, we introduced shape-based screening into our study. This approach is not only fast enough for screening several million compounds but also suitable for referring to the shape of the ligand binding site cavity during the virtual screening. Additionally, in the virtual screening process, to more accurately rank-ordering potential ligands before biological testing, we incorporated single conformer MM-GBSA binding energy calculations, which use an implicit solvent model. This model can rapidly evaluate over 50,000 compounds, taking into account the solvation effect during the screening process while maintaining reasonable performance compared to those obtained after averaging over multiple MD snapshots with explicit water molecules. This results in significant savings in computing time without sacrificing accuracy [42]. Although absolute free-energy calculations using an explicit solvent model offer greater accuracy, they are considerably time-consuming. While the GBSA method is theoretically less accurate than the MM–Poisson–Boltzmann Surface Area (PBSA) method [48], its favorable balance between accuracy and computational efficiency makes it a valuable choice, especially for qualitative analysis [49]. Consequently, using virtual screening, we identified BC-001 as a tool compound, with an IC_50_ value of 3677 nM in cellular assays. Our in silico predictions and in vitro assay results demonstrated good consistency, thus underscoring the potential of BC-001.

We also incorporated MD simulations into the MM-GBSA method to account for the flexibility of the protein and enhance the accuracy of predictions. This integration forms a robust approach for post-MD MM-GBSA analysis. We explain how this method could be used to predict compounds from other research groups retrospectively. In this study, we utilized post-MD MM-GBSA analysis to predict optimal compounds. After analyzing Merck’s compounds, we discovered that introducing a trifluoromethyl group to our scaffold greatly enhanced binding affinity. Our findings showed that compounds containing trifluoromethyl groups were more potent than those without them. In addition, we believe that the hydrazide group may be unstable, which is why it was substituted with several other head groups. We observed that the optimized compound BC-011, which contains an amide and trifluoromethyl group, displayed an IC_50_ of 72.43 nM in the reporter assay. Moreover, BC-011 displayed an IC_50_ of 488.5 nM in the cell viability assay and also decreased the TEAD target gene expression. In conclusion, our study highlights the benefits of integrating FMO analysis and MD simulations, as well as MM-GBSA calculations, into the virtual screening process for identifying hit compounds. This multifaceted approach, which accounts for the solvation effect, provides enhanced reliability and predictive accuracy compared to relying on a single calculation method [43,50].

## 4. Materials and Methods

### 4.1. Protein Structure Preparation

The crystal structures of the human TEAD2 and MGH-CP1 complexes were obtained from the Protein Data Bank (PDB ID: 6CDY). Protein structures were prepared using the Protein Preparation Wizard. All missing loops were filled using Prime and implemented in Maestro (version 2022-4) (both from Schrödinger, LLC, New York, NY, USA, 2022). Hydrogen atoms were added to the complex structure at pH 7.4, and their positions were optimized using PROPKA in Maestro. Restrained energy minimization was performed with an OPLS4 force field, within 0.3 Å root mean square deviation (RMSD).

### 4.2. FMO Calculations

The GAMESS program was used to perform all the FMO calculations [51], wherein the two-body FMO method was used, and each residue in the protein and ligand was defined as a fragment. All input files were prepared using our in-house code, in compliance with hybrid orbital projection scheme fragmentation [52]. As mentioned in our previous report, the two-body FMO method includes 4 steps [30]: fragmentation, one-fragment self-consistent field calculation, two-fragment self-consistent field calculation, and total property evaluation. All residues in the protein were divided at the sp3 bond between the alpha and carbonyl carbon atoms, based on the hybrid orbital projection scheme [52].

We used the second-order Møller–Plesset perturbation theory [53] and polarizable continuum model [54], with a 6-31** basis set.

The pair interaction energies (PIEs) between two fragments were measured, and the energy decomposition of the PIEs between fragments in the FMO calculations was performed using the five energy terms defined in Equation (1).

(1) $${\Delta E}^{int}={\Delta E}^{es}+{\Delta E}^{ex}+{\Delta E}^{ct+mix}+{\Delta E}^{di}+{\Delta G}_{sol}$$

where ΔE^es^ is the electrostatic term, ΔE^ex^ is the exchange-repulsion term, ΔE^ct+mix^ is the charge transfer with a higher-order mixed term, ΔE^di^ is dispersion, and ΔG_sol_ is the solvation energy obtained from the polarizable continuum model. PIE decomposition analysis provides a summation of the decomposed energy terms. A PIE less than −3.0 kcal/mol represents a stable interaction between a ligand and specific residue within 5.0 Å to sort [55].

### 4.3. Shape-Screening

We performed shape-screening in Phase (Maestro, version 2022-4) for shape-based flexible ligand alignments and database screening based on an initial alignment of many similar atom triplets followed by refinement [56]. This resulted in the best-aligned structure overlapping the query structure for each searched molecule. In this study, we prepared type atom- and pharmacophore-based shape data for running the graphics processing unit. The Molport screening database was used (https://www.molport.com/). After shape-screening, we set 2000 compounds as the maximum number of ligands to be saved per query.

### 4.4. Molecular Docking

Molecular docking was conducted to generate binding poses from the results of shape-screening, using Glide (Maestro, version 2022-4). We also generated the binding poses of the reference compounds as query compounds during shape-based screening. The optimized ligands from BC-002 to BC-013 were used to investigate the interactions by using docking simulation. The receptor grid generation module (Maestro) was used to define the molecular docking site. Ligands were docked with extra precision (XP) using Glide on TEAD2 (PDB ID: 6CDY).

### 4.5. MD Simulations

The MD simulations were performed using Desmond in Maestro (version 2022-4). The protein–ligand complexes (PDB IDs: 6CDY, 6UYB, 6UYC, 7ZJP, and 7CNL) were inserted into an orthorhombic box filled with explicit water molecules (TIP3P model) and a buffer distance of 10 Å. An MD simulation was performed using the OPLS4 force field, and the non-bonding interactions were typically calculated using force fields. Ions (Na^+^ and Cl^−^) were added to simulate the physiological concentration of monovalent ions (0.15 M). NPT (a constant number of particles, pressure, and temperature) uses constant temperature (300 K) and pressure (1.01325 bar) as the ensemble class. The default protocol in Desmond was used to reach system equilibration, and a 100 ns simulation was performed for each complex [57]. For each system, three 100 ns replicas were performed by adjusting the random number generator seed values to 2007, 5334, and 7558, respectively (Figures S4, S5 and Table S6). The analysis plots and figures were sketched using the Desmond simulation interaction diagram panel of the Maestro program.

Simulation result data were obtained using a Simulation Interaction Diagram from Maestro (version 2022-4). The RMSD from 0 ns (0 frame) to 100 ns (1000 frame) was used to calculate the average fluctuation in the displacement of a selection of atoms for a particular frame relative to the reference frame. This was measured for all snapshots of the trajectory. The RMSD was calculated using Equation (2),

(2) $${RMSD}_{X}=\sqrt{\frac{1}{N}\sum_{i=1}^{N}{\left({r^{\prime }}_{i}\left(t_{x}\right)\right)-r_{i}(t_{ref}))}^{2}}$$

where N is the number of atoms in the atom selection, t_ref_ is the reference time, and r′ is the position of the selected atoms in frame x, after superimposition on the reference frame, where frame x is recorded at time t_x_. This process was repeated for each frame of the simulation trajectory.

All protein frames were first aligned on the frame 0 backbone, and the protein RMSD was then calculated. The protein α carbon RMSD can provide the protein structural conformation change during simulation. Fluctuations from 1 to 3 Å represent a stable protein. The ligand RMSD indicates the stability of the small molecule in the binding pocket.

### 4.6. MM-GBSA Calculation

MM-GBSA calculations were performed to predict the free binding energies of the receptor and ligand complexes. Prime MM-GBSA calculates the energy of optimized free receptors, free ligands, and a complex using an implicit model called VSGB 2.0 suit [43]. MM-GBSA estimates the relative binding free energy of the protein–ligand complex [50]. The binding free energy ΔG_bind_ [58] was calculated using Equation (3),

(3) $${\Delta G}_{bind}=G_{complex}-G_{ligand}-G_{protein}$$

where ΔG_bind_ is the estimated binding free energy, G_complex_ is the MM-GBSA energy of the minimized complex, G_ligand_ is the MM-GBSA energy of the free ligand, and G_protein_ is the MM-GBSA energy of the apoform of the protein.

**Figure 13:**
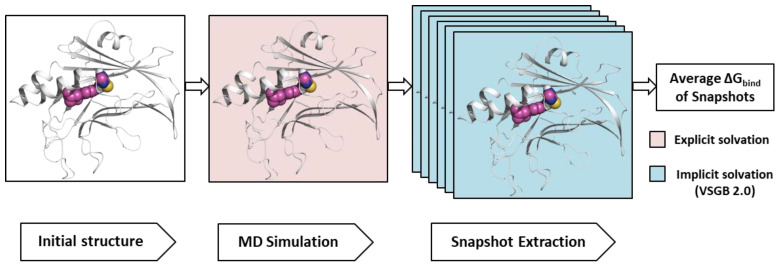
Workflow of the post-MD MM-GBSA analysis. MD, molecular dynamics; MM, molecular mechanics; GBSA, Generalized Born Surface Area.

After the 100 ns simulation, we ran the *themal_mmgbsa.py* script of the Prime/Desmond module in Schrödinger to calculate the average MM-GBSA ΔG_bind_. We obtained snapshots per 10 frames from the trajectory, and Prime MM-GBSA was performed on the complex from each snapshot, as mentioned above [46]. The average ΔG_bind_ value was calculated using Equation (4),

(4) $${\bar{\Delta G}}_{bind}=\frac{1}{n}\sum_{i=1}^{n}{\Delta G}_{i}$$

where n is the number of snapshots, i is the selected frame snapshot, and G_i_ is the ΔG_bind_ of the snapshot MM-GBSA binding energy. We also chose 5, 10, 20, and 50 ns frames to measure the average value of MM-GBSA ΔG_bind_. The protocol for post-MD MM-GBSA analysis is shown in Figure 13.

Gibbs free energy includes enthalpy and entropy, but calculating the latter is time-consuming, involving methods such as the normal-mode analysis method [50]. Therefore, MM-GBSA binding energy was mainly contributed to by enthalpy energy in this study.

### 4.7. General Methods for Compound Synthesis and Analysis

All the reactants, reagents, and solvents were obtained from commercial sources and used without further purification. Except where noted otherwise, the reactions were performed under an inert atmosphere of nitrogen gas using anhydrous solvents. The reactions were monitored using liquid chromatography–mass spectrometry (Agilent 1290 Infinity LC, Agilent 6120 quadrupole LC/MS, Agilent, Santa Clara, CA, USA). Flash chromatography was performed using the CombiFlash^®^ system (Teledyne Isco, St. Lincoln, NE, USA). All nuclear magnetic resonance spectra were collected using a 600 MHz JNM-ECZ600R spectrometer (JEOL, Akishima, Tokyo). Chemical shifts (d in ppm) in the ^1^H spectra were reported relative to the residual solvent signals: 7.26 ppm for chloroform-d.

### 4.8. Synthesis of Compounds

Step a: K_2_CO_3_ (2 eq) was added to a solution of methyl 3-(bromomethyl)-4-methoxybenzoate (1 eq) in DMF (0.1 M). The mixture was stirred at 80 °C for 6 h and then poured into brine and extracted with EA. The combined organic layers were concentrated in vacuo, and the residue was purified using silica gel chromatography (0–50% EA/hexane), to provide a-1 (78% yield) and b-1 (82% yield).

Step b: NaOH (1.2 eq) was added to a solution of a-1 or b-1 in MeOH/water (3:1) (0.1 M). The mixture was stirred at 80 °C for 3 h. The reaction mixture was poured into saturated aqueous ammonium chloride and extracted with EA. The combined organic layers were concentrated in vacuo, and the residue was purified by means of silica gel chromatography (0–10% MeOH/MC) to obtain a-2 (63% yield) and b-2 (78% yield).

Step c: HATU (2 eq), NH_4_Cl (2 eq), and TEA (5 eq) were added to a solution of a-2 or b-2 in DMF (0.1 M). The mixture was stirred at room temperature for 2 h and then poured into brine and extracted with EA. The combined organic layers were concentrated in vacuo, and the residue was purified by means of silica gel chromatography (0–10% MeOH/MC) to obtain a-3 (41% yield) and b-3 (37% yield).

Step d: CDI (1.2 eq) and hydrazine hydrate (2 eq) were added to a solution of BC-008 and BC-012 in THF (0.1 M). The mixture was stirred at room temperature for 3 h and then poured into brine and extracted with EA. The combined organic layers were concentrated in vacuo, and the residue was purified by means of silica gel chromatography (0–10% MeOH/MC) to obtain BC-006 (63% yield) and BC-010 (55% yield).

All steps mentioned above are represented in Figure S6.

### 4.9. Cell Culture and Stable Cell Lines

Stable cell lines, namely MCF7 cell TEAD luciferase cells, were maintained in Dulbecco’s modified Eagle medium (DMEM) containing 10% fetal bovine serum, penicillin, and streptomycin, at 37 °C, in a humidified incubator containing 5% CO_2_. To construct an MCF7-TEAD luciferase stable cell line, MCF7 cells were infected with a TEAD luciferase reporter lentivirus using polybrene (Millipore, Burlington, MA, USA) and then selected with puromycin. The stable cell lines NCI-H229 and NCI-H28 were maintained in Roswell Park Memorial Institute (RPMI) medium.

### 4.10. Luciferase Assay

TEAD luciferase reporter MCF7 cells were seeded into 96-well white plates (3 × 10^4^ cells per well). The medium was replaced with DMEM without fetal bovine serum (FBS; 100 μL per well), and the cells were treated with various concentrations of compounds (0.001–10 μmol/L) for 24 h. Fifty microliters of the medium containing compounds was removed from each well, following which 50 μL of FBS in 10% DMEM was added to the cells and incubated for 6 h. Luciferase activity was measured using the One-Glo^®^ Luciferase Assay Kit from Promega (Madison, WI, USA). A small portion of the medium was removed (60 μL) from each well, and 40 μL of One-Glo^®^ Luciferase assay buffer containing reagent was added in its place. Luminescence was measured using a VICTOR^®^ Nivo™ multimode microplate reader (PerkinElmer, Waltham, MA, USA) and normalized to the results of the vehicle.

### 4.11. mRNA Expression

H226 cells were seeded at a density of 1 × 10^5^ cells/well into a 12-well plate. After treatment with various concentrations (1, 5, and 10 μM) of the compounds, the cells were incubated at 37 °C for 24 h. RNA extraction was performed using the RNeasy Plus Mini Kit (QIAGEN, Hilden, Germany), as per the manufacturer’s protocol. Extracted RNA was quantified using a NanoDrop™ One Microvolume UV–Vis Spectrophotometer (Thermo Fisher, Waltham, MA, USA). cDNA was synthesized using 1000 ng of quantified RNA and an iScript™ cDNA Synthesis Kit (Bio-Rad, Hercules, CA, USA). qRT-PCR was carried out using PowerUp™ SYBR™ Green Master Mix (Thermo Fisher) and primers (hCTGF-F: CCAATGACAACGCCTCCTG, hCTGF-R: TGGTGCAGCCAGAAAGCTC, hCYR61-F: AGCCTCGCATCCTATACAACC, hCYR61-R: TTCTTTCACAAGGCGGCACTC, hβ-actin-F: GCCGACAGGATGCAGAAGGAGATCA, hβ-actin-R: AAGCATTTGCGGTGGACGATGGA), with the results normalized to those of the vehicle.

### 4.12. Cell Viability Assay

Cells were seeded into 96-well plates (1 × 10^3^ cells per well) and changed to RPMI with 1% FBS, and then treated with various concentrations (0.0001–10 μmol/L) of compounds (MGH-CP1, BC-010, BC-011, and BC-013) for 144 h. The medium was replaced with 1% FBS + RPMI containing compounds every 3 d. Cell viability was measured using the CellTiter-Glo^®^ 2.0 Cell Viability Assay Kit from Promega. Before the viability was measured, the drug treatment medium was removed and replaced with 50 μL of FBS-free RPMI and 50 μL of CellTiter-Glo^®^ 2.0 reagent. The cells with medium replacement were incubated for 1 h at room temperature, to prevent exposure to light. Luminescence was measured using a VICTOR^®^ Nivo™ multimode microplate reader (PerkinElmer) and normalized to the results of the vehicle.

## 5. Conclusions

In this study, we explored the TEAD lipidation modulators using a comprehensive and appropriate structure-based drug design approach, which deepened our understanding of the characteristics of TEAD’s binding site. Our study extensively analyzed TEAD inhibitors, encompassing computational studies and experimental validation. These findings have important implications in the field of TEAD inhibition and drug discovery, providing valuable insights that could be pivotal in the development of new therapeutics for various cancers, especially those targeting the Hippo signaling pathway.

## Figures and Tables

**Table 1:** SAR of hit compound BC-001.

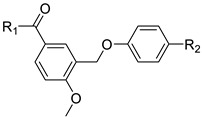				
Compound	R_1_	R_2_	IC_50_ (nM)	MMGBSA dG (kcal/mol)
BC-006	─NH─NH_2_	H	1515	−63.38
BC-007	─NH_2_	H	5608	−58.98
BC-008	─OH	H	>10,000	−52.85
BC-009	─OCH_3_	H	2607	−60.81
BC-010	─NH─NH_2_	─CF_3_	768.2	−66.51
BC-011	─NH_2_	─CF_3_	72.43	−68.86
BC-012	─OH	─CF_3_	8904	−56.77
BC-013	─OCH_3_	─CF_3_	416.9	−62.87

## Data Availability

Protein structures used in this manuscript were downloaded from the Protein Data Bank (See Figure 1); five structures were mainly used (PDB IDs: 6CDY, 6UYB, 6UYC, 7ZJP, and 7CNL). The FMO code was incorporated into the open-source General Atomic and Molecular Electronic Structure System (GAMESS version 30 September 2022, R2), which is a general ab initio quantum chemistry package. Protein preparation, molecular docking simulation, shape-based screening, MD simulation, MM-GBSA calculation, and post-MD MM-GBSA analysis were performed using Schrödinger software (Maestro version 2022-4).

## Supplementary Materials

The following supporting information can be downloaded at: https://www.mdpi.com/article/10.3390/ijms25105358/s1.
