# Supplementary material for: Leveraging the Fragment Molecular Orbital and MM-GBSA Methods in Virtual Screening for the Discovery of Novel Non-Covalent Inhibitors Targeting the TEAD Lipid Binding Pocket
Source: Int J Mol Sci. 2024 May 14;25(10):5358. doi: 10.3390/ijms25105358 (PMC11121470; doi:10.3390/ijms25105358)
Supplement: Supplementary file 1 [file ijms-25-05358-s001.zip › ijms-2973477-supplementary.pdf]

Leveraging the Fragment Molecular Orbital and MM-GBSA Methods in Virtual Screening for the Discovery of Novel Non-Covalent Inhibitors Targeting the TEAD Lipid Binding Pocket

Jongwan Kim [1,2,*,†], Haiyan Jin [2,3,†], Jinhyuk Kim [3,4], Seon Yeon Cho [4], Sungho Moon [4], Jianmin Wang [3], Jiashun Mao [3] and Kyoung Tai No [2,3,4,5,*]

[1] Department of Structural Biology, St. Jude Children's Research Hospital, Memphis, TN 38105, USA

[2] Bioinformatics and Molecular Design Research Center (BMDRC), Incheon 21983, Republic of Korea; haiyanjin@yonsei.ac.kr

[3] The Interdisciplinary Graduate Program in Integrative Biotechnology & Translational Medicine, Yonsei University, Incheon 21983, Republic of Korea; yetyet123@yonsei.ac.kr (J.K.); jmwang113@hotmail.com (J.W.); jiashun_mao@yonsei.ac.kr (J.M.)

[4] Baobab AiBIO Co., Ltd., Incheon 21983, Republic of Korea; sycho@baobabaibio.com (S.Y.C.); shmoon@baobabaibio.com (S.M.)

[5] Department of Biotechnology, Yonsei University, Seoul 03722, Republic of Korea

* Correspondence: myulchi@yonsei.ac.kr (J.K.), ktno@yonsei.ac.kr (K.T.N.)

† These authors contributed equally to this work.

Index	Shape type	Query number	Similarity normalization	ROC	RIE	Number of ranked actives
1	Pharmacophore	28	Maximum	0.99	16.89	28
2	Pharmacophore	7	Maximum	0.46	8.67	13
3	Typed atom	28	Maximum	0.99	17.22	28
4	Typed atom	7	Maximum	0.43	8.02	12
5	Pharmacophore	28	query structure	0.78	13.52	22
6	Pharmacophore	7	query structure	0.28	5.32	8
7	Pharmacophore	28	Minimum	0.21	3.73	6
8	Pharmacophore	7	Minimum	0.04	0.68	1
9	Pharmacophore	28	Screen structure	0.21	3.83	6
10	Pharmacophore	7	Screen structure	0.07	1.37	2

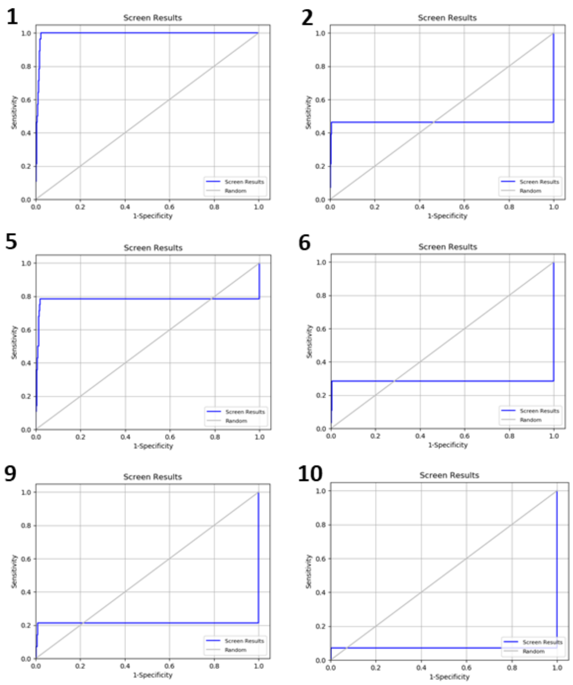

Figure S1. Enrichment calculation results of shape-based screening test.

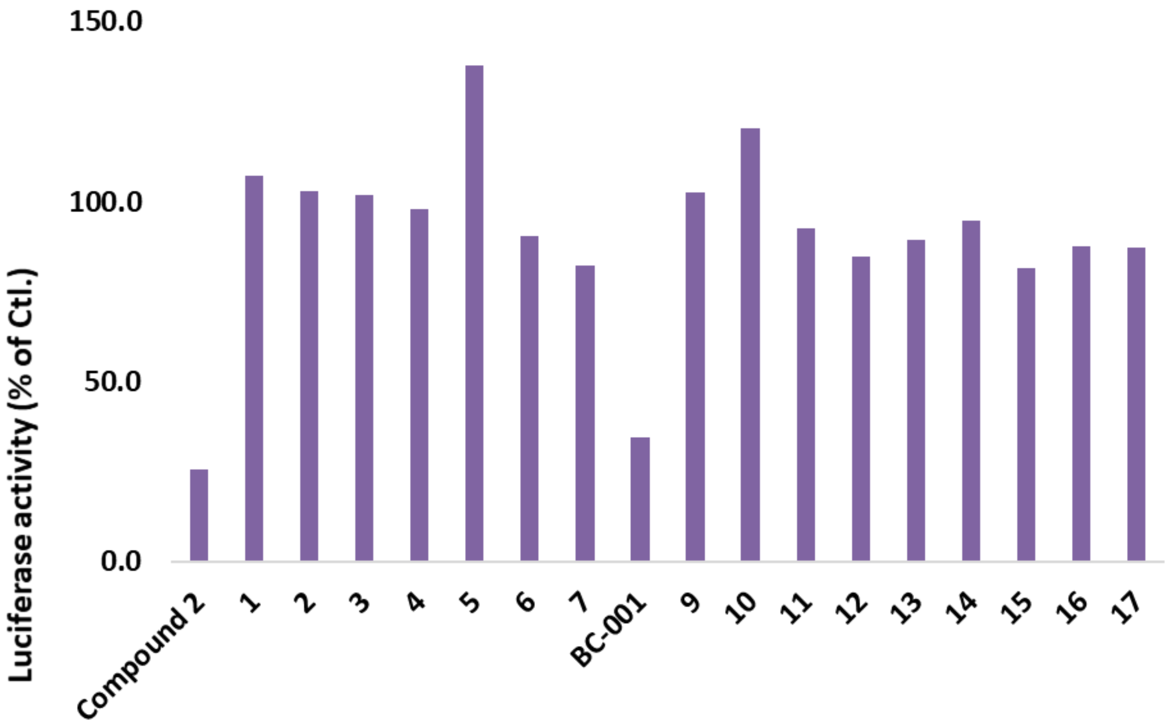

Figure S2. The TEAD luciferase reporter assay of 17 virtual hits.

TEAD reporter luciferase activity was observed in MCF7 cells treated with 10 μM of Compound 2 and 17 virtual hits.

A

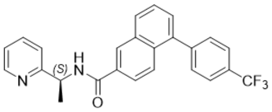

VT – 104

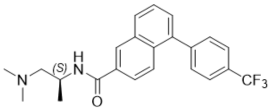

EX64

Compound	5ns (kcal/mol)	10ns (kcal/mol)	20ns (kcal/mol)	50ns (kcal/mol)	100ns (kcal/mol)	RMSD Stable (kcal/mol)	IC50 (μM)
VT−104	−86.02	−84.95	−83.39	−80.73	−80.81	−83.25	0.01
EX64	−76.72	−72.57	−72.59	−73.15	−71.75	−73.15	1~3

−86.02 (kcal/mol) −76.72 (kcal/mol)

B

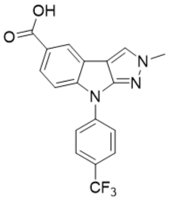

MSC−4106

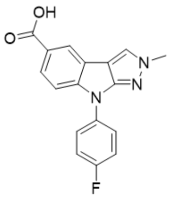

Merck_20

Compound	5ns (kcal/mol)	10ns (kcal/mol)	20ns (kcal/mol)	50ns (kcal/mol)	100ns (kcal/mol)	RMSD Stable (kcal/mol)	IC50(μM)
MSC−4106	-64.37	-64.2	-63.92	-63.73	-63.79	-63.68	0.014<br>1.9
Merck_20	-60.84	-60.18	-59.44	-59.2	-59.51	-59.91	1~10

−64.37 (kcal/mol) −59.20 (kcal/mol)

Figure S3. Retrospective prediction of post-MD MM-GBSA ΔG of Vivace and Merck compounds. We chose the 5ns, 10ns, 20ns, 50ns, RMSD more stable frame region from the 100 ns trajectory to calculate the post-MD MM-GBSA ΔG values. A. Vivace and B. Merck compounds. MD, molecular dynamics; MM, molecular mechanics; GBSA, Generalized Born Surface Area; RMSD, root mean square deviation.

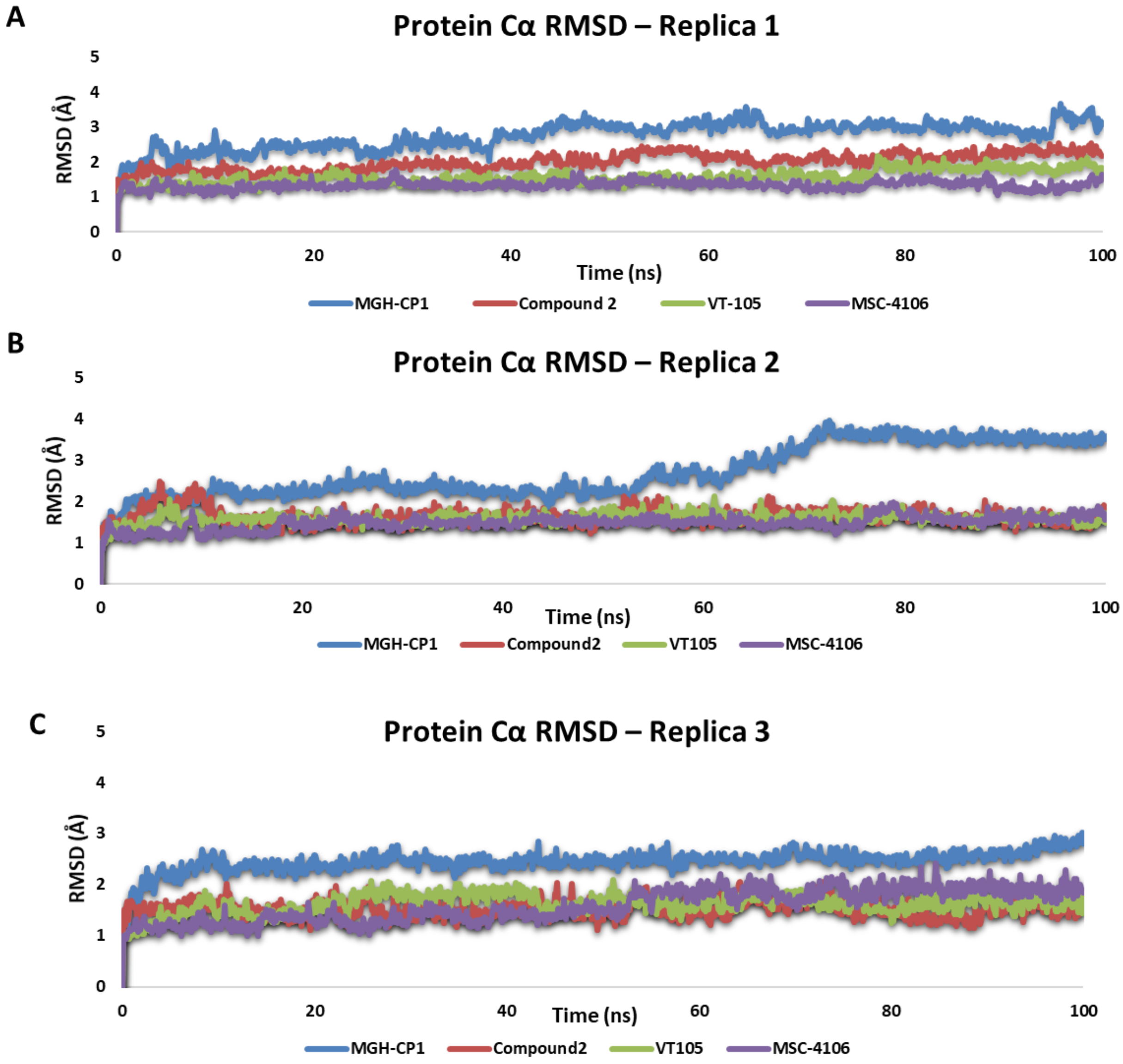

Figure S4. Protein Cα RMSD calculations of 3 different replicas.

A. Protein Cα RMSD of replica 1, also shown in Figure 5A. B. Protein Cα RMSD of replica 2. C. Protein Cα RMSD of replica 3.

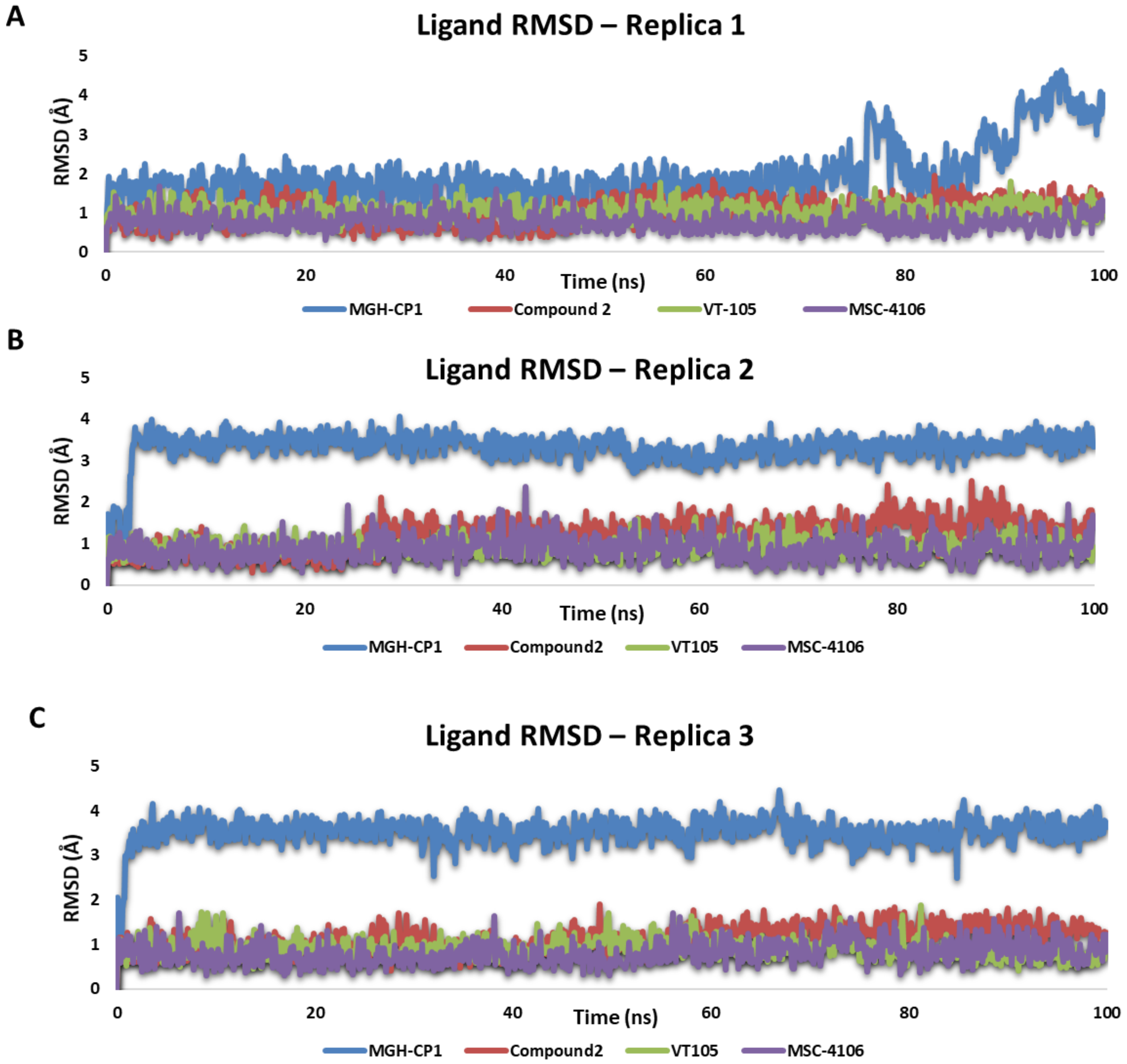

Figure S5. Ligand RMSD calculations of 3 different replicas

A. Ligand RMSD of replica 1, also shown in Figure 5B. B. Ligand RMSD of replica 2. C. Ligand RMSD of replica 3.

Synthetic scheme

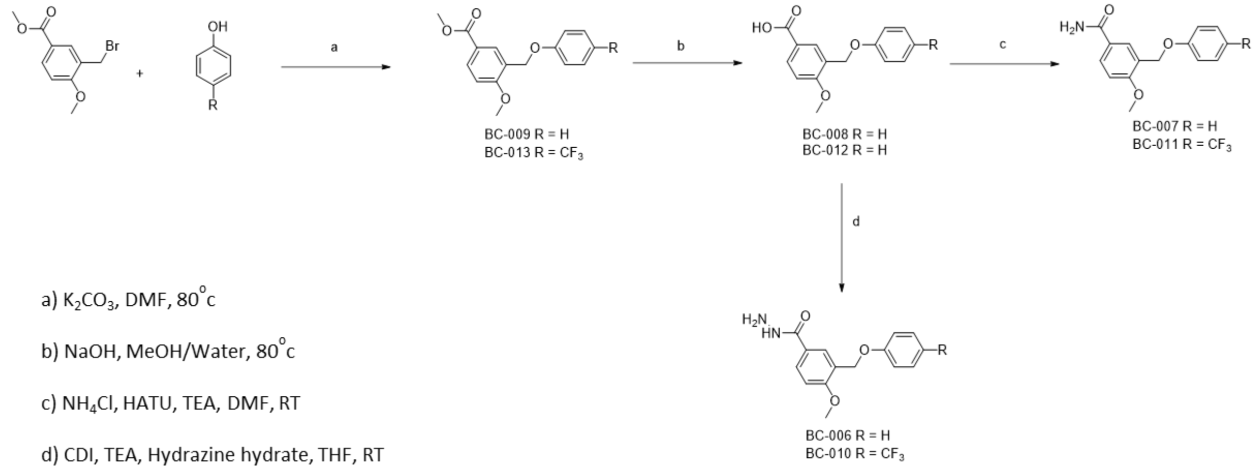

Figure S6. The synthetic scheme of compounds in the paper.

Table S1. PIEDA of MGH-CP1 and TEAD2 complex. All energies are in kcal/mol. The calculation was conducted at the FMO-MP2/6-31G**/PCM level.

Residue	ΔE^{int}	ΔE^{es}	ΔE^{ex}	ΔE^{ct+mix}	ΔE^{di}	ΔG_{sol}
Ile408	−3.79	−0.798	2.75	−1.436	−4.456	0.149
Leu383	−3.038	−1.249	5.961	−1.568	−6.215	0.033
Lys357	−6.385	−1.324	2.334	−1.05	−4.225	−2.12
Met379	−5.148	−1.509	1.33	−1.015	−4.143	0.188
Phe428	−5.063	−2.67	7.243	−1.69	−7.992	0.047
Ser345	−4.552	−3.644	0.299	−0.715	−1.46	0.967
Val329	−4.998	−7.116	12.293	−2.592	−7.162	−0.421

Solvent: water

Table S2. PIEDA of Compound2 and TEAD2 complex. All energies are in kcal/mol. The calculation was conducted at the FMO-MP2/6-31G**/PCM level.

Residue	ΔE^{int}	ΔE^{es}	ΔE^{ex}	ΔE^{ct+mix}	ΔE^{di}	ΔG^{sol}
Cys380	−11.701	−14.899	13.607	−3.761	−6.485	−0.162
Gln410	−4.995	−3.442	1.077	−1.131	−2.47	0.971
Ile408	−5.019	−0.982	2.905	−1.374	−5.606	0.038
Leu383	−3.517	−3.5	8.17	−1.754	−6.354	−0.079
Lys357	−6.395	−4.381	0.329	−0.391	−1.429	−0.523
Phe233	−3.074	−2.347	6.792	−1.587	−5.853	−0.079
Phe428	−4.875	−1.219	3.589	−1.688	−5.715	0.158
Ser345	−4.941	−2.955	0.643	−0.794	−2.024	0.189

Solvent: water

Table S3. PIEDA of MSC-4106 and TEAD1 complex. All energies are in kcal/mol. The calculation was conducted at the FMO-MP2/6-31G**/PCM level.

Residue	ΔE^{int}	ΔE^{es}	ΔE^{ex}	ΔE^{ct+mix}	ΔE^{di}	ΔG_{sol}
Cys359	−34.712	−31.926	6.611	−4.75	−5.173	−1.617
Ile366	−4.218	−1.68	1.362	−0.888	−2.639	−2.928
Leu369	−3.217	−1.501	0.098	−0.397	−0.623	−3.843
Lys336	−103.158	−105.175	3.44	−3.812	−5.013	5.136
Met362	−11.756	−7.957	6.413	−2.116	−7.884	−2.547
Phe221	−5.854	−2.876	4.225	−1.439	−6.674	−1.893
Phe224	−3.224	−3.474	−0.001	0.037	−0.285	−4.343
Phe239	−5.301	−2.167	0.527	−0.845	−2.166	−3.444
Phe407	−6.264	−2.703	4.785	−1.985	−6.851	−1.885
Thr324	−9.289	−4.816	1.13	−0.792	−2.093	−4.966
Val240	−6.207	−2.708	2.756	−1.387	−5.023	−2.478

Solvent: water

Table S4. PIEDA of VT-105 and TEAD3 complex. All energies are in kcal/mol. The calculation was conducted at the FMO-MP2/6-31G**/PCM level.

Residue	ΔE^{int}	ΔE^{es}	ΔE^{ex}	ΔE^{ct+mix}	ΔE^{di}	ΔG_{sol}
Ala232	−3.032	−1.753	2.194	−0.846	−2.846	0.218
Cys368	−13.387	−11.272	5.631	0.868	−8.099	−0.515
Glu344	−4.374	−4.699	0.493	−0.452	−1.659	1.942
Lys345	−4.331	−1.714	10.307	−2.607	−9.223	−1.094
Met367	−8.886	−5.247	13.005	−4.804	−11.406	−0.434
Met371	−9.577	−5.489	5.072	−1.989	−6.853	−0.318
Phe248	−4.643	−0.756	2.387	−1.643	−4.532	−0.098
Phe416	−4.711	−3.816	8.2	−1.774	−7.382	0.06
Tyr230	−5.79	−4.954	4.599	−1.45	−4.292	0.307

Solvent: water

Table S5. PIEDA of BC-001 and TEAD2 complex. All energies are in kcal/mol. The calculation was conducted at the FMO-MP2/6-31G**/PCM level.

Residue	ΔE^{int}	ΔE^{es}	ΔE^{ex}	ΔE^{ct+mix}	ΔE^{di}	ΔG_{sol}
Cys380	−16.161	−18.946	14.835	−4.764	−6.804	−0.483
Leu383	−7.186	−3.462	5.204	−1.952	−6.673	−0.303
Lys357	−4.372	1.883	2.921	−1.128	−3.73	−4.318
Met379	−15.937	−17.403	15.795	−5.773	−9.539	0.984
Phe233	−4.05	−2.166	3.856	−1.301	−4.423	−0.017
Phe428	−5.765	−2.713	3.947	−1.624	−5.434	0.06
Ser345	−3.967	−2.888	0.05	−0.317	−0.666	−0.146
Tyr426	−3.028	−1.372	2.001	−0.995	−2.759	0.096

Solvent: water

Table S6. The post-MD MM-GBSA analysis of three different replicas.

Compound	5ns					10ns				
	Replica1	Replica2	Replica3	Mean	SD	Replica1	Replica2	Replica3	Mean	SD
BC−001	−73.46	−61.35	−68.83	−67.88	6.11	−68.64	−62.63	−69.83	−67.03	3.86
BC−002	−67.38	−68.88	−42.18	−59.48	15.00	−65.99	−64.72	−46.89	−59.20	10.68
BC−003	−71.58	−67.69	−63.10	−67.46	4.24	−68.34	−64.91	−59.59	−64.28	4.41
BC−004	−72.43	−72.43	−83.20	−76.02	6.22	−71.66	−71.66	−83.98	−75.77	7.12
BC−005	−61.97	−61.97	−56.36	−60.1	3.24	−63.39	−62.25	−58.47	−61.37	2.58
BC−006	−63.01	−62.52	−55.86	−60.46	3.99	−62.90	−60.15	−58.92	−60.66	2.04
BC−007	−64.80	−58.76	−53.49	−59.02	5.66	−66.00	−60.05	−57.33	−61.13	4.44
BC−008	−55.37	−50.34	−56.71	−54.14	3.36	−56.78	−50.66	−56.13	−54.52	3.36
BC−009	−63.47	−65.68	−63.37	−64.17	1.30	−66.56	−64.01	−63.73	−64.77	1.56
BC−010	−72.21	−66.34	−63.02	−67.19	4.65	−72.63	−57.07	−63.77	−64.49	7.80
BC−011	−66.37	−66.04	−64.88	−65.76	0.79	−67.60	−66.46	−65.10	−66.39	1.25
BC−012	−60.96	−66.03	−59.13	−62.04	3.58	−63.51	−62.18	−56.94	−60.87	3.48
BC−013	−70.16	−70.98	−59.04	−66.72	6.67	−68.96	−70.51	−60.31	−66.60	5.50
VT−105	−88.93	−91.3	−90.31	−90.18	1.19	−88.04	−91.82	−88.10	−89.32	2.16
VT−104	−91.02	−81.37	−85.67	−86.02	4.83	−90.99	−81.02	−82.84	−84.95	5.31
EX64	−77.01	−74.84	−78.32	−76.72	1.76	−77.47	−67.30	−72.95	−72.57	5.10
MSC−4106	−65.32	−65.02	−62.75	−64.37	1.41	−64.82	−64.91	−62.87	−64.20	1.15
Merck_20	−61.94	−60.97	−59.62	−60.84	1.17	−61.53	−59.25	−59.76	−60.18	1.20

SD, Standard deviation

Table S6. The post-MD MM-GBSA analysis of three different replicas (continued).

Compound	20ns					50ns				
	Replica1	Replica2	Replica3	Mean	SD	Replica1	Replica2	Replica3	Mean	SD
BC−001	−65.94	−64.73	−70.77	−67.15	3.19	−68.97	−64.96	−69.11	−67.68	2.36
BC−002	−64.80	−60.46	−58.42	−61.23	3.26	−62.28	−59.18	−55.93	−59.13	3.17
BC−003	−68.48	−64.49	−65.68	−66.22	2.05	−65.57	−64.37	−63.63	−64.52	0.98
BC−004	−71.98	−71.98	−78.78	−74.24	3.93	−68.36	−68.36	−76.14	−70.95	4.49
BC−005	−63.01	−63.01	−60.70	−62.24	1.34	−59.62	−59.62	−58.95	−59.39	0.39
BC−006	−65.06	−57.90	−62.55	−61.84	3.63	−66.62	−56.49	−62.59	−61.90	5.10
BC−007	−67.50	−58.38	−60.07	−61.98	4.85	−68.25	−55.41	−55.58	−59.75	7.37
BC−008	−56.47	−51.69	−52.92	−53.70	2.48	−57.50	−52.94	−50.93	−53.79	3.36
BC−009	−67.09	−65.10	−62.17	−64.79	2.47	−67.45	−59.44	−62.39	−63.09	4.05
BC−010	−72.45	−60.71	−60.58	−64.58	6.82	−70.67	−62.97	−63.87	−65.84	4.21
BC−011	−68.12	−66.42	−65.42	−66.66	1.36	−69.29	−66.23	−68.65	−68.05	1.61
BC−012	−63.87	−60.25	−55.77	−59.96	4.06	−62.37	−58.16	−56.58	−59.04	2.99
BC−013	−69.62	−66.05	−62.30	−65.99	3.66	−70.66	−57.06	−61.62	−63.11	6.92
VT−105	−89.27	−91.61	−89.58	−90.15	1.27	−90.32	−91.30	−91.10	−90.91	0.52
VT−104	−91.59	−78.57	−80.02	−83.39	7.13	−91.41	−72.50	−78.27	−80.73	9.69
EX64	−78.82	−68.75	−70.20	−72.59	5.44	−82.22	−67.30	−69.92	−73.15	7.97
MSC−4106	−64.26	−64.79	−62.72	−63.92	1.08	−63.44	−63.91	−63.85	−63.73	0.25
Merck_20	−60.89	−57.79	−59.63	−59.44	1.56	−60.84	−58.22	−58.53	−59.20	1.43

SD, Standard deviation

Table S6. The post-MD MM-GBSA analysis of three different replicas (continued).

Compound	100ns					RMSD Stable				
	Replica1	Replica2	Replica3	Mean	SD	Replica1	Replica2	Replica3	Mean	SD
BC−001	−69.53	−65.16	−68.79	−67.83	2.34	−71.55	−68.82	−63.82	−68.06	3.92
BC−002	−61.48	−57.98	−60.04	−59.84	1.76	−66.18	−61.50	−61.51	−63.06	2.70
BC−003	−63.73	−61.50	−61.85	−62.36	1.20	−62.16	−64.18	−67.58	−64.64	2.74
BC−004	−69.16	−69.16	−74.11	−70.81	2.86	−70.51	−69.31	−74.61	−71.48	2.78
BC−005	−58.71	−58.71	−55.49	−57.64	1.86	−60.78	−55.72	−60.71	−59.07	2.90
BC−006	−66.74	−60.42	−62.97	−63.38	3.18	−67.30	−59.37	−65.55	−64.07	4.17
BC−007	−66.95	−54.19	−55.81	−58.98	6.95	−67.73	−58.29	−58.90	−61.64	5.28
BC−008	−59.69	−49.62	−49.23	−52.85	5.93	−59.66	−49.33	−46.82	−51.94	6.81
BC−009	−65.46	−56.77	−60.18	−60.81	4.38	−65.91	−59.23	−59.26	−61.47	3.85
BC−010	−70.66	−63.31	−65.54	−66.51	3.77	−70.17	−69.72	−67.22	−69.04	1.59
BC−011	−70.36	−67.10	−69.13	−68.86	1.65	−70.99	−68.03	−69.74	−69.58	1.48
BC−012	−60.08	−54.65	−55.58	−56.77	2.91	−57.13	−49.53	−52.70	−53.12	3.82
BC−013	−70.38	−57.24	−61.00	−62.87	6.77	−70.94	−55.76	−56.52	−61.07	8.55
VT−105	−90.08	−91.31	−91.78	−91.06	0.88	−91.69	−91.21	−91.06	−91.32	0.33
VT−104	−91.16	−75.11	−76.15	−80.81	8.98	−91.45	−79.16	−79.14	−83.25	7.10
EX64	−82.28	−66.64	−66.33	−71.75	9.12	−81.93	−71.45	−66.06	−73.15	8.07
MSC−4106	−63.89	−63.89	−63.58	−63.79	0.17	−64.72	−63.38	−62.94	−63.68	0.93
Merck_20	−60.40	−59.38	−58.77	−59.51	0.83	−60.27	−60.06	−59.40	−59.91	0.45

SD, Standard deviation
